# The oncogenic role of MUC12 in RCC progression depends on c‐Jun/TGF‐β signalling

**DOI:** 10.1111/jcmm.15515

**Published:** 2020-06-28

**Authors:** Sheng‐Lin Gao, Rui Yin, Li‐Feng Zhang, Si‐Min Wang, Jia‐Sheng Chen, Xing‐Yu Wu, Chuang Yue, Li Zuo, Min Tang

**Affiliations:** ^1^ Department of Urology The Affiliated Changzhou No.2 People's Hospital of Nanjing Medical University Changzhou China; ^2^ Center for Reproductive Medicine Shandong Provincial Hospital Affiliated to Shandong University Jinan China; ^3^ Changzhou Third People's Hospital Changzhou China; ^4^ Department of Urology The First Affiliated Hospital of Nanjing Medical University Nanjing China

**Keywords:** c‐Jun, invasion, MUC12, renal cell carcinoma, TGF‐β1

## Abstract

Renal cell carcinoma (RCC) is a common kidney cancer worldwide. Even though current treatments show promising therapeutic effectiveness, metastatic RCC still has limited therapeutic options so that novel treatments were urgently needed. Here, we identified that MUC12 was overexpressed in RCC patients and served as poor prognostic factor for RCC progression. Overexpression of MUC12 increased RCC cell growth and cell invasion while deficiency of MUC12 exerted opposite effects on RCC cells. Mechanistic dissection demonstrated that MUC12‐mediated RCC cell growth and cell invasion were dependent of TGF‐β1 signalling because they could be blocked in the presence of TGF‐β1 inhibitor. Moreover, the regulation of TGF‐β1 by MUC12 relied on the transactivation of c‐Jun. MUC12 promoted the recruitment of c‐Jun on the promoter of TGF‐β1, leading to its transcription. Importantly, knockdown of c‐Jun also attenuated MUC12‐mediated TGF‐β1 induction and RCC cell invasion. In summary, our study defines the role of MUC12 in RCC progression and provides rational to develop novel targeted therapy to battle against RCC.

## 1 INTRODUCTION

Renal cell carcinomas (RCCs) are most lethal type of kidney cancer, and its incidence is continuously rising.^1^, ^2^ RCCs can be classified into four major histological cell types, and clear cell type (ccRCC) accounts for approximately 80% of RCC cases.^3^ Gender difference, genetic factors and life style are common risk factors for ccRCC.^4^, ^5^ Although current treatments benefit patients' lives a lot, the median survival of advanced RCC is only 26 months. Therefore, novel therapies are urgently being developed for the better treatment of RCC. Large‐scale genomic analyses of RCC revealed that the majority of RCC was caused by VHL mutation.^6^, ^7^ Classically, VHL is E3 ligase for hypoxia‐inducible factors (HIF‐1α and HIF‐2α) and its inactivation increases HIF levels in RCC tumours.^8^, ^9^ The well‐known target of HIF signalling is VEGF family, which plays central role in the development of neo‐angiogenesis and tumour metastasis. Therefore, targeting the angiogenetic signalling pathway becomes an ideal therapeutic strategy for RCC patients. As a first‐line medicine for metastatic RCC, sunitinib is designed to suppress angiogenesis by inhibiting receptor tyrosine kinases (RTKs) in both RCC cells and endothelial cells.^10^, ^11^

Mucin family belongs to glycoprotein and mainly locates in cell membrane.^12^, ^13^, ^14^ The dysregulation of mucin proteins has been frequently observed in malignant cancers.^14^ The participation of mucin proteins into cancer progression relies on its capacity to transduce intracellular signallings. Previous studies have demonstrated that MUC1 is overexpressed in several cancers including breast cancer and pancreatic cancer.^14^, ^15^ MUC1 not only serves as a cancer biomarker but also functions to regulate several biological events of cancer including proliferation, invasion, immune‐therapy and drug resistance.^15^ However, as one of mucin family member, MUC12 is rarely investigated, especially in RCC.

The transforming growth factor‐β (TGF‐β) signalling is involved in considerably biological events including cell survival, cell differentiation, immune response and cancer development.^16^ Upon the TGF‐β binding, TGF‐β receptor is phosphorylated and activated, which in turn promotes the phosphorylation of SMAD2/SMAD3. The phosphorylated SMAD2/SMAD3 is then complexed with SMAD4 and translocation to the nucleus to regulate a plethora of genes.^17^, ^18^ Initially, TGF‐β signalling is considered to play tumour suppressing role by inhibiting cell proliferation and inducing apoptosis.^19^ However, as tumour cells progress, they develop mechanisms to switch TGF‐β signalling as a driving force for cancer progression. Indeed, mounting evidence suggests that TGF‐β signalling plays central role in the process of epithelial‐mesenchymal transition (EMT), an early process facilitates tumour cells to metastasize to distant organs.^20^

In this study, we found that MUC12 was overexpressed in RCC patients compared with normal kidney tissues. Knockdown of MUC12 suppressed RCC cell growth and cell invasion while induction of MUC12 in RCC cells revealed the opposite phenotypes. Mechanistically, MUC12 triggered TGF‐β1 activation via increasing its reliable transcription factor c‐Jun. Inhibition of TGF‐β1 or c‐Jun could attenuate MUC12‐induced RCC cell growth and cell invasion. Overall, our data strengthen the oncogenic role of MUC12 in RCC progression and provide strong rational to develop novel therapy to better suppress advanced RCC.

## 2 METHODS AND MATERIALS

### 2.1 Human clinical samples

All tumour tissues and adjacent normal tissues were obtained from Department of Urology, The Affiliated Changzhou No. 2 People's Hospital of Nanjing Medical University, from 2016 to 2018. We performed IHC on 4% formaldehyde‐fixed tissues. Twenty‐four pairs of fresh tumour tissues and the corresponding peritumoural tissues were used for qRT‐PCR and Western blot (WB) assays.

### 2.2 Cell culture

HK2, SW839, 786‐O, OSRC A498 and Caki‐1 were obtained from the American Type Culture collection (ATCC, Rockville, MD, USA). Cells were cultured in DMEM media containing 1% penicillin and streptomycin, supplemented with 10% foetal bovine serum (FBS) and maintained in a 5% (v/v) CO2 humidified incubator at 37°C.

### 2.3 Lentivirus generation

pWPI‐based plasmids or PLKO‐based shRNA plasmids (20 µg), psPAX2 packaging plasmid (10 µg) and pMD2.G envelope plasmid (10 µg) were cotransfected into 293T cells using the standard calcium phosphate transfection method. 48 hours later, lentivirus supernatant was collected by 0.45 µm filter and infected RCC cells in the presence of 8 µg/mL polybrene. 1 µg/mL puromycin was added to select PLKO‐based shRNA positive RCC cells.

### 2.4 Quantitative real‐time PCR

A 1 μg of total RNA was subjected to reverse transcription using Superscript III Transcriptase (Invitrogen, Grand Island, NY). Quantitative real‐time PCR (qRT‐PCR) was performed in a Bio‐Rad CFX96 machine with SYBR green to determine the interested mRNAs. GAPDH mRNA was used as internal control. The primers used in this study were listed in Table S3.

### 2.5 MTT assay

Renal cell carcinoma cells with gene manipulation were seeded into 24‐well plate at the density of 5000. Cells at different time‐points (days 0, 2, 4 and 6) were incubated with 5 µg/mL 3‐(4, 5‐dimethylthiazolyl)‐2 and 5‐diphenyltetrazolium bromide (MTT) for 2 hours. Then, medium was removed and OD570 value was measured.

### 2.6 Colony formation assay

RCC cells with gene manipulation were seeded into 6‐well plate at the density of 500. 2 weeks later, cells were fixed by cold methanol and stained with 0.1% crystal violet.

### 2.7 Invasion assay

A 8 µm transwells were pre‐coated with diluted Matrigel (1:10 with serum‐free medium) and dried for 2 hours in the incubator. Infected RCC cells were seeded into the upper transwell chamber at the density of 1 × 10^5^. 20% serum medium was added in the lower chamber to attract the cells. One day later, the invaded cells were fixed by cold methanol and stained with 0.1% crystal violet. Images were captured by microscopy.

### 2.8 Western blot analysis

Renal cell carcinoma human samples or cells were lysed in RIPA buffer. 40 μg proteins were loaded for separation on 8%‐12% SDS/PAGE gel. Then, proteins were transferred onto PVDF membranes (Millipore, Billerica, MA, USA). After being blocked with 10% milk for 1 hour, the membranes were blotted with specific primary antibodies at 4°C overnight. Membranes were washed with 0.1% Tween‐20 TBS and incubated with HRP‐conjugated secondary antibodies for another one hour. Blots were visualized using ECL system (Thermo Fisher Scientific, Rochester, NY, USA). The following primary antibodies were used in this study: MUC12 (sc‐26372, Santa Cruz, CA, USA); GAPDH (sc‐47724, Santa Cruz, CA, USA); N‐cadherin (sc‐8426, Santa Cruz, CA, USA); E‐cadherin: (sc‐59987, Santa Cruz, CA, USA); Snail‐1 (sc‐271977, Santa Cruz, CA, USA); vimentin (sc‐6260, Santa Cruz, CA, USA); ZO‐1: (sc‐33725, Santa Cruz, CA, USA); TGF‐β1 (sc‐130348, Santa Cruz, CA, USA); p‐SMAD3 (sc‐11769, Santa Cruz, CA, USA); and c‐Jun (sc‐166540, Santa Cruz, CA, USA).

### 2.9 Analysis of TCGA data set

KIRC raw data were downloaded from TCGA database for the analysis of MUC12 expression between RCC patients (n = 523) and normal kidney tissues (n = 100). For gene set enrichment analysis (GSEA) of single gene, GSEA (v4.0.3) was used to perform, and MUC12 median level was used as the cut‐off criterion.

### 2.10 Immunohistochemistry (IHC) staining

Antigen retrieval was performed by boiling the deparaffinized and rehydrated sections in a citrate buffer (pH 6.0) for 30 minutes. Then, sections were treated with a peroxidase blocking buffer for 20 minutes at RT. After incubation with the blocking buffer (3%BSA + 3% milk in PBS) for 1 hour at RT, sections were blotted with MUC12 antibody (ab121777, Abcam) at 4°C overnight. After 3 times wash, sections were then incubated with biotin‐labelled secondary antibody for another 1 hour and MUC12 signal was determined by DAB staining.

### 2.11 Chromatin immunoprecipitation (ChIP)

ChIP assay was performed as described in previous report.^21^ Protein‐DNA complexes were cross‐linked by formaldehyde for 10 minutes and then quenched by 125 mmol/L glycine for 10 minutes. Genomic DNAs were sonicated to an average of 500 bp size. After centrifugation, the DNA‐protein complexes were precipitated with c‐Jun antibody overnight at 4 ºC. Next, pre‐cleared A/G beads were added for the purification of c‐Jun binding DNAs.

### 2.12 Luciferase assay

RCC cells were cotransfected with pGL3‐TGFβ‐WT or pGL‐TGFβ‐MUT (200 ng/well), c‐Jun siRNAs (10 mmol/L) or negative siRNAs (10 mmol/L) and pRL‐TK (5 ng/well) using Lipofectamine 3000 according to the manufacturer's instructions. 48 hours later, cells were lysed and the luciferase activity was detected by the dual luciferase assay using pRL‐TK as the internal control. Each reading was performed in triplicate.

### 2.13 Animal studies

The animal protocol was approved by the Institutional Animal Use and Care Committee of Nanjing Medical University. As for xenografts group, mice were inoculated subcutaneously with 1 × 10^6^/100 μL 786‐O cells stably transfected with shMUC12. The implanted tumour volume of each mouse was monitored every 7 days, and tumour weight was calculated after 5 weeks when mice were sacrificed.

### 2.14 Statistics

SPSS 23.0 software (IBM, USA) was used to conduct statistical analyses. Results of the experiment were shown as the mean ± SD. Differences in different groups were analysed by Student's t test or one‐way ANOVA. Correlations between different parameters were analysed using a Spear man rank test. Statistical tests were performed using GraphPad Prism 8.0. *P* < 0.05 was considered statistically significant. The detailed results were *P < 0.05, **P < 0.01 and ***P < 0.001.

## 3 RESULTS

### 3.1 Analysis of TCGA data set showed that MUC12 levels were positively correlated with RCC progression

**FIGURE 1:**
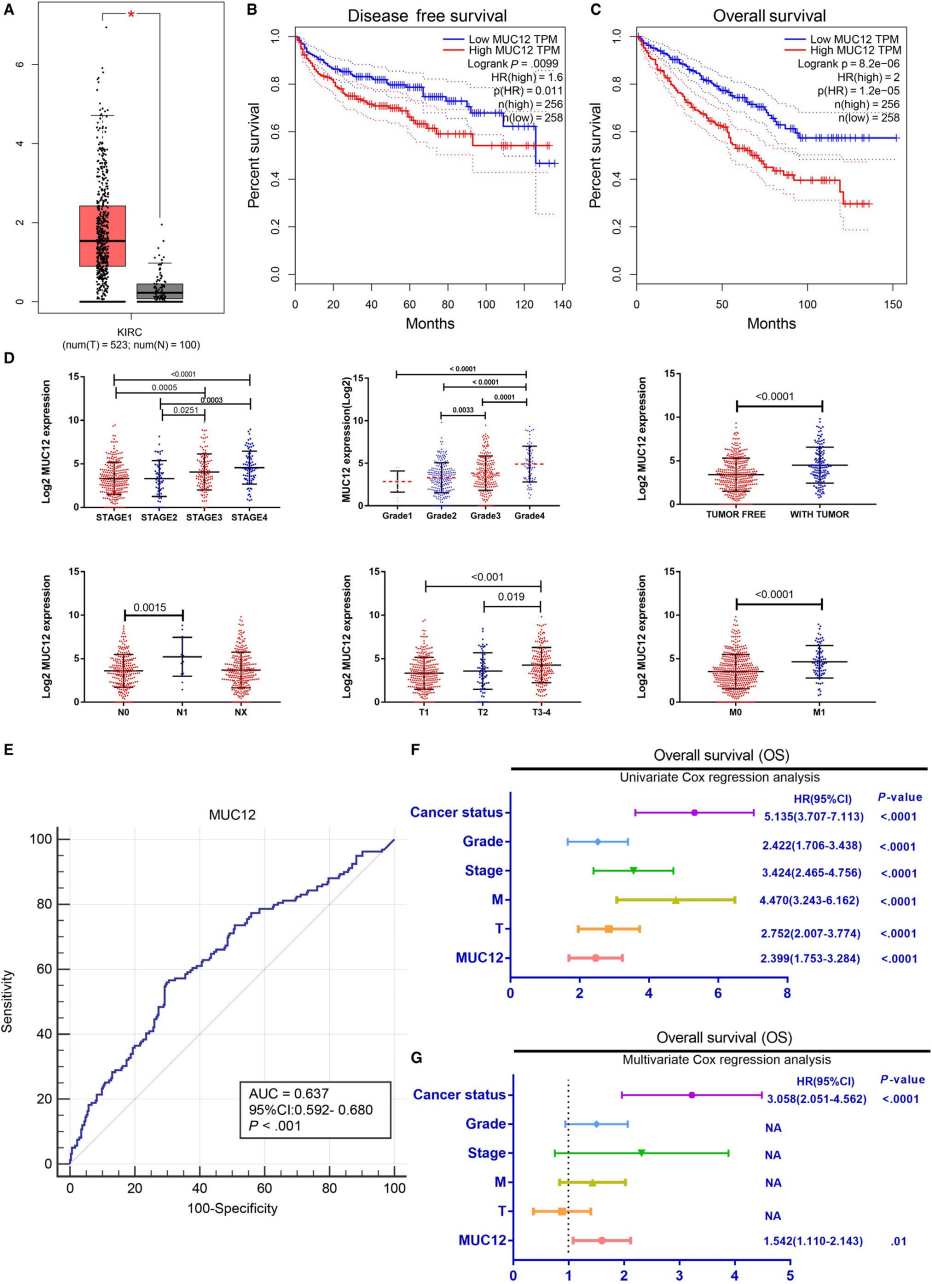
Analysis of TCGA data set showed that MUC12 levels were positively correlated with RCC progression. A, MUC12 was up‐regulated in RCC patients compared to normal kidney tissues. B‐C, MUC12 was poor prognostic factor for RCC overall survival (B) and disease‐free survival (C). D, MUC12 was up‐expression in high clinical stage, pathological grade, tumour recurrence and tumour metastasis RCC. E, ROC curve revealed that MUC12 could be as a worse prognostic indicator (95% CI: 0.592‐0.680, *P* < 0.001) for RCC patients. F‐G, Univariate (F) and multivariate (G) analysis showed that MUC12 was an independent risk factor for overall survival, tumour recurrence, tumour grade and cancer metastasis. *P < 0.05

To investigate the potential role of MUC12 in RCC carcinogenesis and development, we first analysed its expression levels based on RNA‐seq from TCGA database. Analysis showed that expression levels of *MUC12* were significantly up‐regulated in RCC patients (n = 523) compared with normal kidney tissues (n = 100) (Figure 1A). In addition, Kaplan‐Meier overall survival (OS) and disease‐free survival (DFS) analyses all suggested that *MUC12* served as a poor prognostic factor for RCC patients (Figure 1B‐C). Importantly, *MUC12* expression levels were also strongly correlated with clinical stage, pathological grade, tumour recurrence and tumour metastasis (Figure 1D), showing that higher *MUC12* levels were observed in more advanced RCC patients. Besides, the ROC curve revealed that *MUC12* could be as a worse prognostic indicator (95% CI: 0.592‐0.680, *P* < 0.001) for RCC patients (Figure 1E). Overall, *MUC12* was an independent risk factor for worse overall survival, tumour recurrence, grade and metastasis according to univariate or multivariate analysis (Figure 1F‐G and Table S1,S2). Together, all these data indicate that *MUC12* may serve as tumour‐promoting factor in RCC patients.

Moreover, analyses of The Cancer Genome Atlas (TCGA) database showed that *MUC12* was significantly high expression in various types of human cancer, such as kidney renal papillary cell carcinoma, oesophageal carcinoma, pancreatic adenocarcinoma, rectum adenocarcinoma, stomach adenocarcinoma, thymoma, head and neck squamous cell carcinoma (Figure 1A) and *MUC12* overexpression correlated with poor prognosis in human cancers, including lung squamous carcinoma, mesothelioma, adenoid cystic carcinoma, uterine carcinosarcoma and uveal melanoma (Figure 1B‐F), which further suggests that *MUC12* may play an oncogenic role in development and progression of various human cancer types.

### 3.2 Experimental examination of MUC12 in RCC patients

**FIGURE 2:**
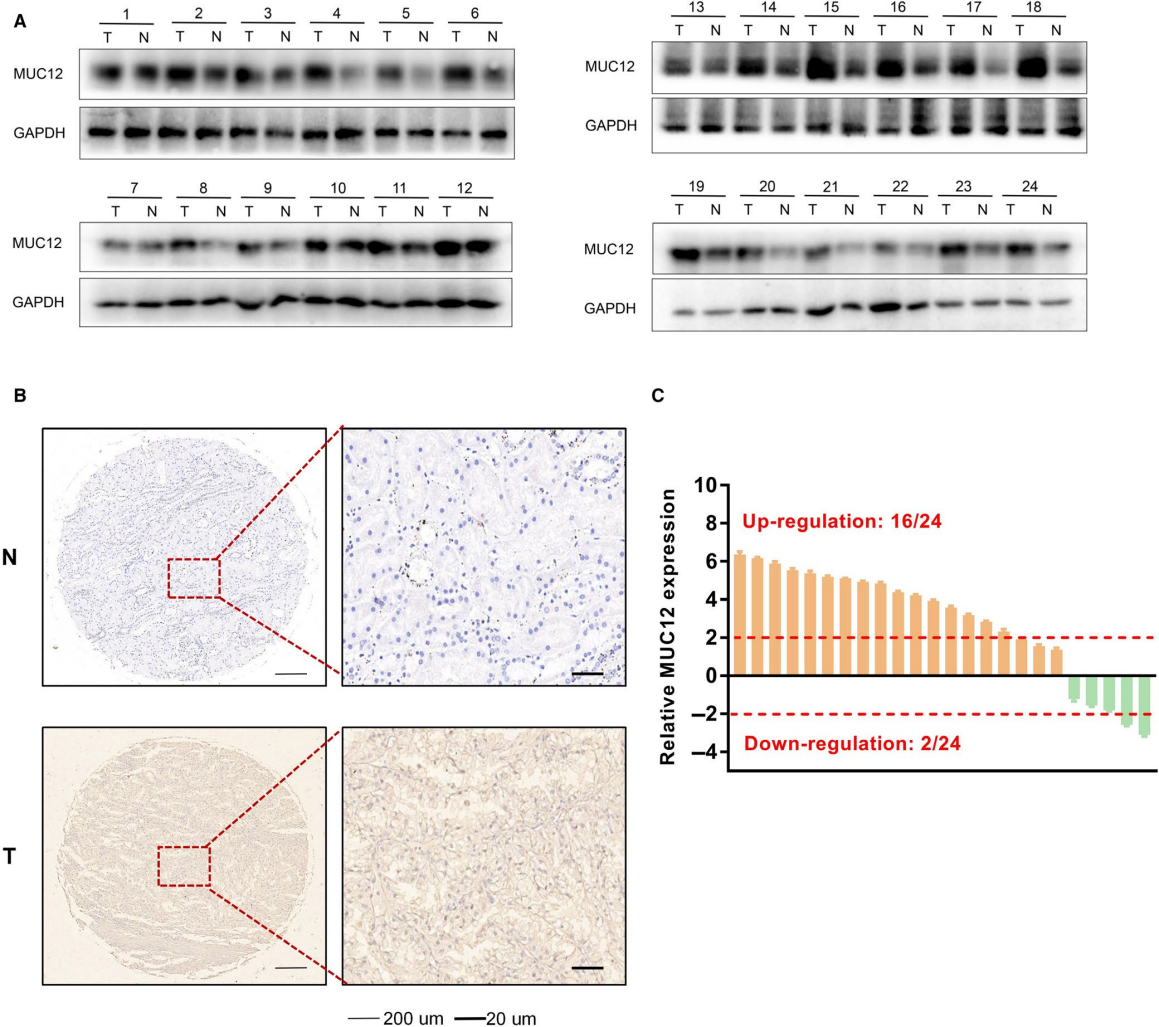
Experimental examination of MUC12 in RCC patients. A, Western blotting analysis of MUC12 in human RCC samples compared to normal kidney tissues. GAPDH was used as loading control. B, Left, representative images of immunohistochemistry staining of MUC12 in human samples compared to normal kidney tissues. Right, a statistical analysis of IHC staining of MUC12. C, QPCR analysis of MUC12 mRNA levels in human RCC samples compared to normal kidney tissues. MUC12 mRNA levels were normalized to GAPDH mRNA levels

To verify the above RNA‐seq data, we collected RCC patients as well as the corresponding normal tissues and examined MUC12 expression levels. Consistent with online analyses, data displayed that MUC12 expression levels were evidently elevated in human RCC samples (n = 24) when compared to normal kidney tissues (n = 24) (Figure 2A), assayed by Western blotting. Immunohistochemistry staining of MUC12 also confirmed that its levels were indeed overexpressed in RCC patients compared with normal kidney tissues (Figure 2B). Additionally, mRNA detection by RT‐PCR showed that 16/24 RCC patients had an up‐regulation of MUC12 while only 2/24 normal kidney tissues selectively expressed higher levels of MUC12 (Figure 2C). Taking together, we speculate that MUC12 plays an oncogenic role in RCC progression.

### 3.3 MUC12 promoted RCC cell growth

**FIGURE 3:**
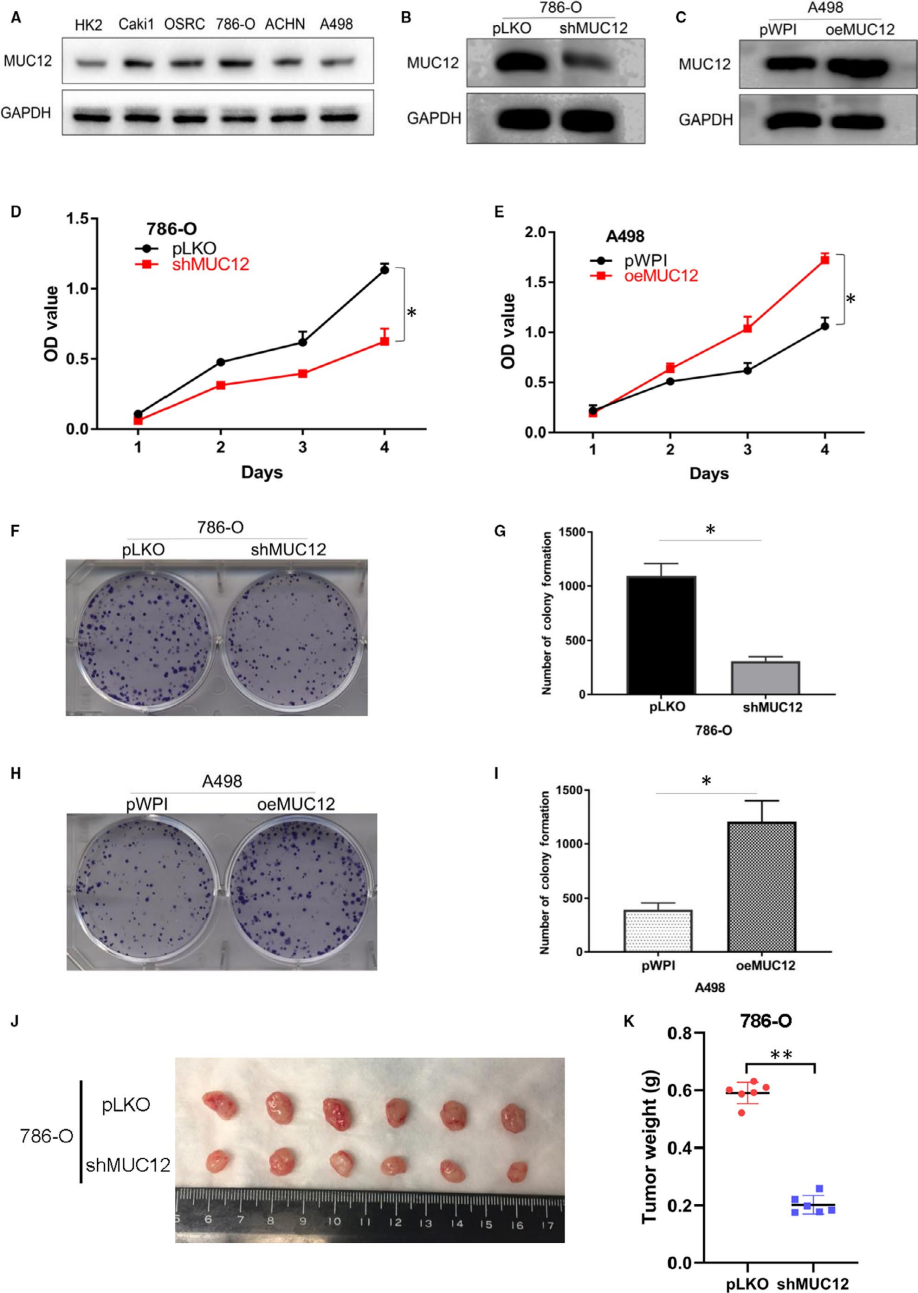
MUC12 promoted RCC cell growth. A, MUC12 expression levels in different RCC cell lines. GAPDH was used as loading control. B, Knockdown efficiency of MUC12 in 786‐O cells. GAPDH served as internal control. C, Confirmation of overexpressed MUC12 in A498 cells. GAPDH was internal control. D, MTT assay showed that knockdown of MUC12 remarkably suppressed cell growth of 786‐O cells. E. MTT assay revealed that MUC12 induction increased cell growth of A498 cells. F‐G. MUC12 depletion by shRNAs decreased colony‐forming ability of 786‐O cells. F, representative images of colonies. G, statistical analysis of F. H‐I, Overexpression of MUC12 increased colony‐forming ability of A498 cells. H, representative images of colonies. I, statistical analysis of H. J, The gross of tumours in shMUC12 and control groups. K, Analysis of tumour weight of xenograft tumours. *P < 0.05; **P < 0.01

To test whether MUC12 is a causal factor for RCC progression, we first compared its expression levels in different RCC cell lines. Result showed that A498 expressed a relatively low level of MUC12 while 786‐O had a relatively high level of MUC12 (Figure 3A), as a comparison to other RCC cell lines. We then knocked down MUC12 in 786‐O and overexpressed MUC12 in A498 (Figure 3B‐C), and tested whether manipulation of MUC12 had any biological functions in RCC cells. Data showed that knockdown of MUC12 in 786‐O cells considerably suppressed their cell growth (Figure 3D), assayed by MTT. Consistently, overexpression of MUC12 in A498 caused a higher growth rate compared to vector bearing cells (Figure 3E). Similarly, 2‐week colony formation assay also confirmed that knockdown of MUC12 in 786‐O cells reduced the colony number while induction of MUC12 in A498 increased the colony number compared to their corresponding controls (Figure 3F‐I). To evaluate the MUC12 function in vivo, the stabilized shMUC12‐786‐O cells and pLKO‐786‐O cells as control were subcutaneously implanted into nude mice (1 × 10^6^ cells per mouse, 6 mice per group). As expected, the tumour volumes of shMUC12 group exhibited smaller compared to the pLKO group at 5 weeks (Figure 3J). Besides, the linear curve also recorded that knocking down MUC12 dramatically suppressed the average weight (Figure 3K) of tumours in nude mice. Together, these results indicate that MUC12 acts as a tumour supporting factor to promote RCC cell growth.

### 3.4 MUC12 enhanced RCC cell invasion via promoting epithelial‐mesenchymal transition (EMT) process

**FIGURE 4:**
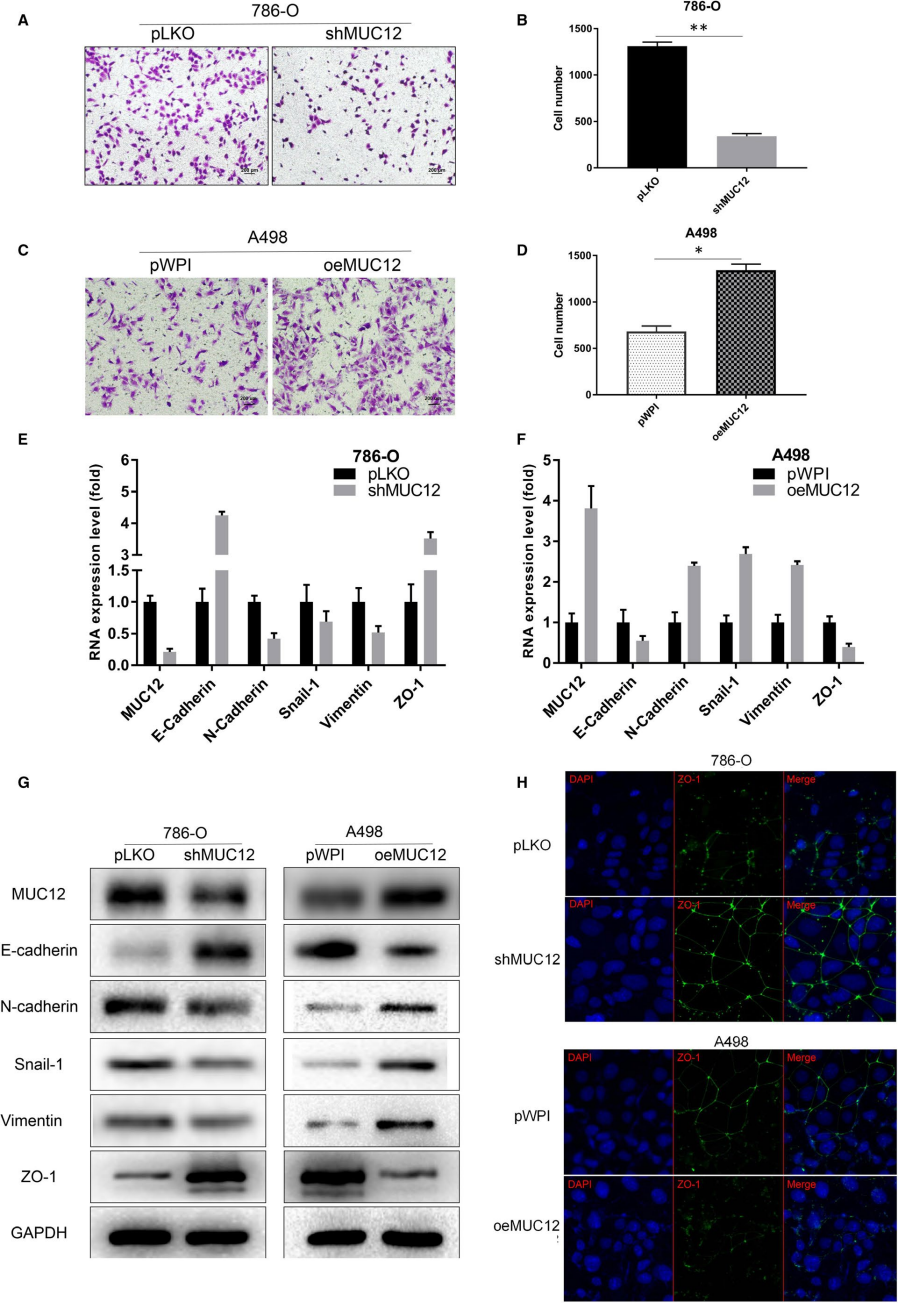
MUC12 enhanced RCC cell invasion via promoting epithelial‐mesenchymal transition (EMT) process. A‐B. Knockdown of MUC12 suppressed cell invasion of 786‐O cells. A, representative invaded cells. B, statistical analysis of A. C‐D, Introduction of MUC12 into A498 cells promoted their invasive abilities. C, representative invaded cells. D, statistical analysis of C. E, Real‐time qPCR demonstrated that MUC12 reduction in 786‐O cells increased E‐cadherin and ZO‐1 while decreased other EMT‐related genes at the mRNA levels. Expression levels of genes were normalized to GAPDH mRNA levels. F, Real‐time qPCR displayed that MUC12 induction in A498 cells reduced E‐cadherin and ZO‐1 while up‐regulated other EMT related genes at the mRNA levels. Expression levels of genes were normalized to GAPDH mRNA levels. G, Western blotting confirmed contribution of MUC12 to EMT‐related markers in both 786‐O and A498 cells. GAPDH was served as loading control. H, Immunofluorescent staining of ZO1 after manipulation of MUC12 in 786‐O and A498 cells. *P < 0.05; **P < 0.01

The ability of cell invasion is another essential hallmark for cancers. To explore whether MUC12 contributes to cell invasion of RCC cells, we applied Matrigel‐based transwell assay to monitor RCC invasive ability before and after MUC12 manipulation. Data revealed that depletion of MUC12 by shRNAs significantly ablated cell invasive ability of 786‐O cells (Figure 4A‐B). In contrast, introduction of MUC12 into A498 cells dramatically elevated their invasive ability (Figure 4C‐D). Given the fact that EMT plays a critical role in the process of cancer invasion and metastasis, we determined to monitor EMT by measuring its related markers. Consistent with phenotype, a deregulation of N‐cadherin, Snail‐1 and vimentin while an up‐regulation of E‐cadherin and ZO‐1 was clearly seen in MUC12‐deficient 786‐O cells (Figure 4E). In contrast, an increase of N‐cadherin, Snail‐1and Vimentin while reduction of E‐cadherin and ZO‐1 was observed in MUC12 expressing A498 cells (Figure 4F). Western blotting analyses also confirmed the above findings (Figure 4G). In addition, ZO‐1 detection by immunofluorescent staining also verified that ZO‐1 signals were reversely correlated with MUC12 levels (Figure 4H). All these findings demonstrate that MUC12 promotes EMT of RCC cells to increase their invasive capacity.

### 3.5 Inhibition of TGF‐β1 attenuated MUC12‐mediated RCC cell growth and cell invasion

**FIGURE 5:**
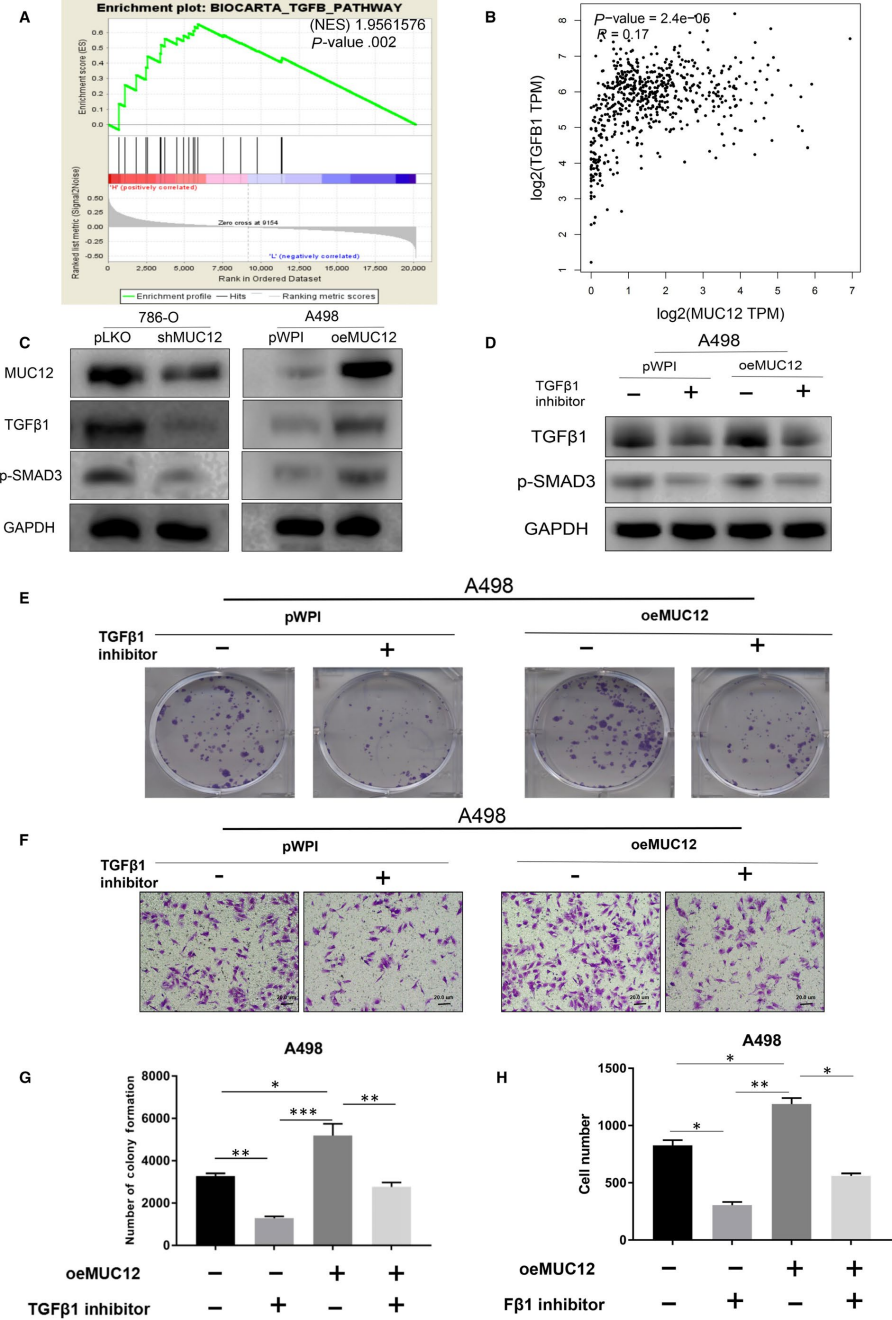
Inhibition of TGF‐β1 attenuated MUC12‐mediated RCC cell growth and cell invasion. A, GSEA analysis of TCGA data set revealed that TGF‐β1 signalling was activated in MUC12 higher group. B, A positive correlation between MUC12 and TGF‐β1 was observed in human RCC samples based on TCGA dataset. *P* < 0.001, *r* = 0.17. C, Knockdown of MUC12 decreased TGF‐β1 levels as well as p‐Smad3 in 786‐O cells while overexpression of MUC12 in A498 cells boosted TGF‐β1 levels as well as p‐Smad3. GAPDH served as internal control. D, Inhibition of TGF‐β1 signalling by its specific inhibitor could reverse MUC12‐mediated induction of TGF‐β1 levels and p‐Smad3 in A498 cells. GAPDH was loading control. E,G, Inhibition of TGF‐β1 signalling by its specific inhibitor blocked MUC12‐mediated RCC cell growth. E, representative images of colonies; G, a statistical analysis of E. F,H, Inhibition of TGF‐β1 signalling by its specific inhibitor attenuated MUC12‐mediated RCC cell invasion. F, representative images of invaded cells; H, a statistical analysis of G. *P < 0.05; **P < 0.01; ***P < 0.001

To find the underlying mechanism by which MUC12 promotes RCC progression, we performed single‐gene set enrichment analysis (GSEA) using the MUC12 median level as a cut‐off. Result exhibited that the activation of TGF‐β1 signalling, a central signalling involved in cancer growth and migration, was observed in MUC12 high group (Figure 5A). In addition, TCGA data set also showed that MUC12 was positively correlated with TGF‐β1 at mRNA level (Figure 5B, *P* < 0.001, *r* = 0.17). Therefore, we sought to examine whether MUC12 could directly affect TGF‐β1 signalling. Expectedly, we found that MUC12 depletion by shRNAs could decrease TGF‐β1 as well as the phosphorylated Smad3 (p‐Smad3) in 786‐O cells (Figure 5C, left). On the contrary, overexpression of MUC12 in A498 cells had strong capacity to increase TGF‐β1 and p‐Smad3 (Figure 5C, right), suggesting the biological functions of MUC12 in RCC cells may rely on TGF‐β1 signalling. To end this, we used TGF‐β1 inhibitor, which was confirmed in Figure 5D by examination of TGF‐β1 and p‐Smad3, to test whether it could attenuate MUC12 mediated cell growth and cell invasion in RCC cells. Data showed that TGF‐β1 inhibitor indeed could reverse MUC12 induced cell growth (Figure 5E,G) and cell invasion (Figure 5F,H) in A498 cells. Collectively, all these results support the notion that MUC12 promotes RCC progression via activating TGF‐β1 signalling.

### 3.6 MUC12‐mediated RCC cell growth was dependent of c‐Jun

**FIGURE 6:**
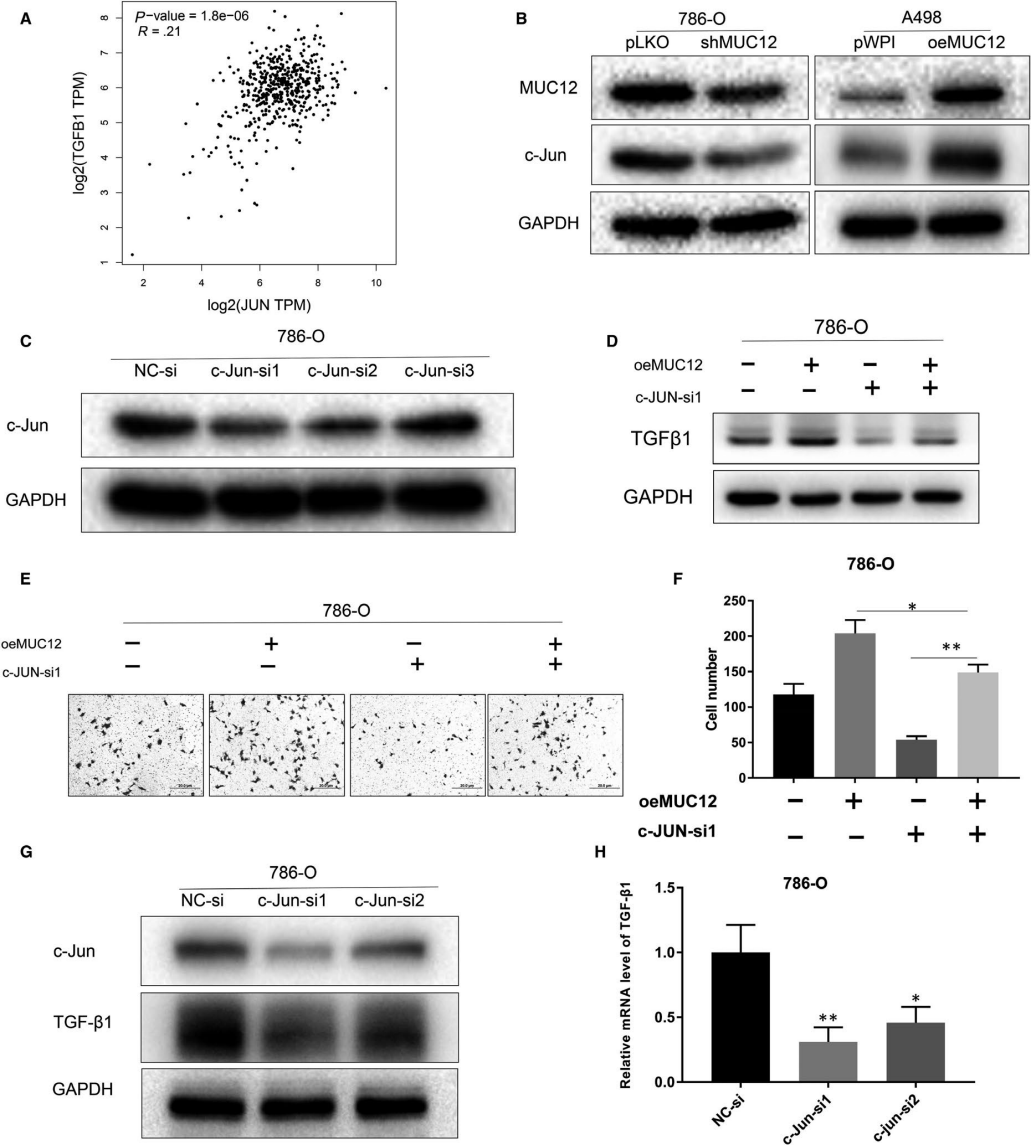
MUC12‐mediated RCC cell progression was dependent of c‐Jun. A, A positive correlation between c‐Jun and TGF‐β1 was observed in human RCC samples based on TCGA data set. B, Knockdown of MUC12 reduced the expression levels of c‐Jun in 786‐O cells while overexpression of MUC12 elevated c‐Jun in A498 cells. GAPDH was internal control. C, Knockdown efficiency of c‐Jun by siRNAs in 786‐O cells. GAPDH was used as loading control. D, Knockdown of c‐Jun attenuated MUC12‐mediated up‐regulation of TGF‐β1. GAPDH served as internal control. E,F, Knockdown of c‐Jun blocked MUC12 induced cell invasion of 786‐O cells. E, representative images of invaded cells. F, a statistical analysis of invaded cells. G, Knockdown of c‐Jun reduced TGF‐β1 protein levels. GAPDH was loading control. H, Knockdown of c‐Jun decreased TGF‐β1 mRNA levels. TGF‐β1 mRNA levels were normalized to GAPDH mRNA levels. *P < 0.05; **P < 0.01

c‐Jun as a transcription factor has been reported to regulate TGF‐β1 at the transcriptional level. Next, we sought to examine whether c‐Jun was involved in MUC12 induced RCC cell growth and cell invasion. Indeed, we found that there was a strongly positive correlation between TGF‐β1 and c‐Jun (Figure 6A, *P* < 0.001, *r* = 0.21) according to the analysis from TCGA data set. Expectedly, knockdown of MUC12 in 786‐O cells could reduce c‐Jun protein levels while overexpression of MUC12 in A498 cells displayed the opposite result (Figure 6B). Importantly, knockdown of c‐Jun by two independent siRNAs, whose efficiency was confirmed in Figure 6C by Western blotting, could reverse MUC12‐mediated induction of TGF‐β1 levels (Figure 6D) as well as MUC12‐mediated increase of RCC cell invasion (Figure 6E). Taken together, these data suggest that c‐Jun was required for MUC12 to activate TGF‐β1 signalling and to promote RCC development.

### 3.7 Activation of TGF‐β1 by MUC12 relied on the transactivation of c‐Jun

**FIGURE 7:**
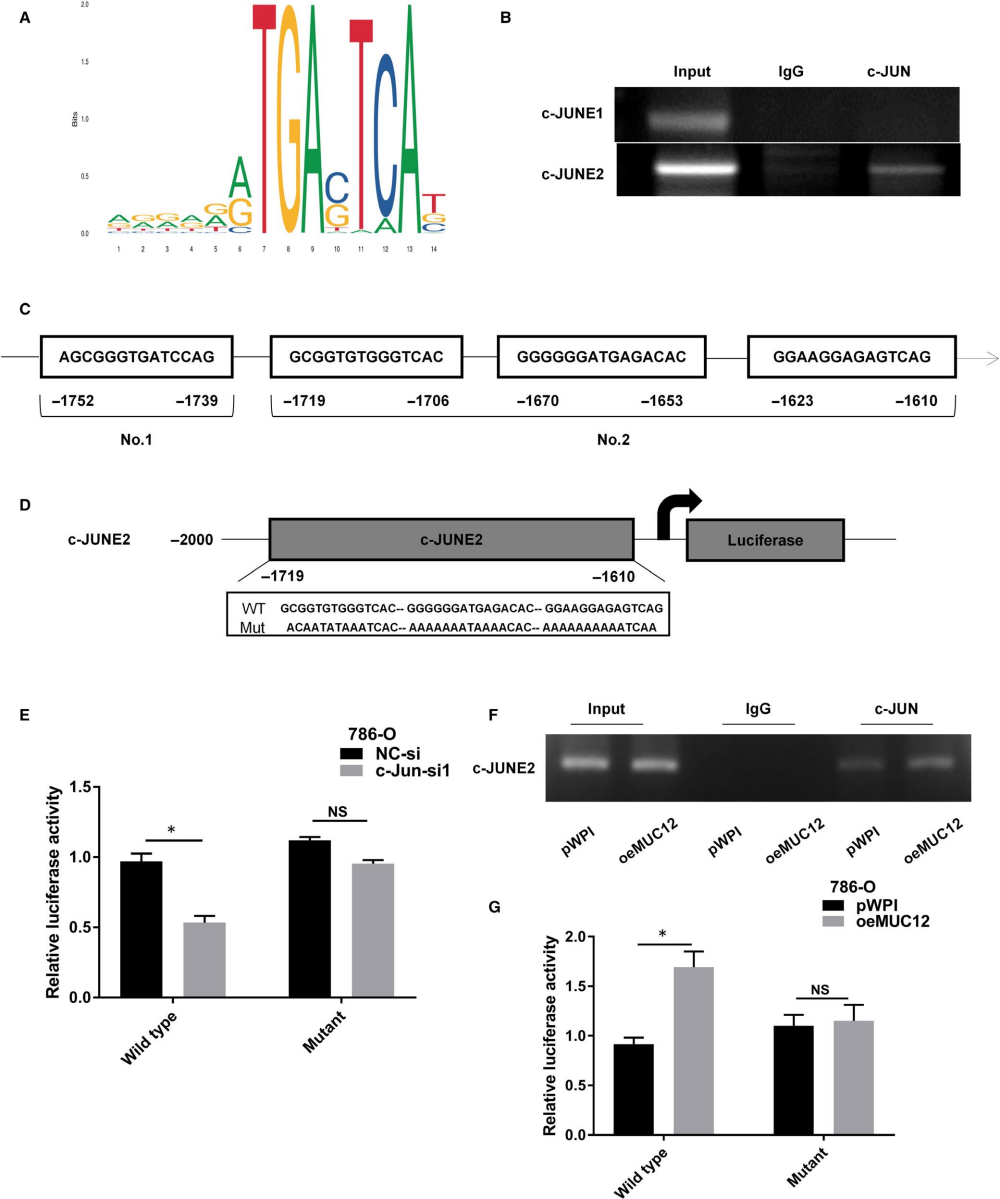
Activation of TGF‐β1 by MUC12 relied on the transactivation of c‐Jun. A,B, There are four potential c‐Jun binding sites in the promoter region of TGF‐β1 according to online prediction. C, ChIP assay showed that c‐Jun could directly bind to c‐JUNE2 but not c‐JUNE1. D, Cartoon was drawn to compare the wild‐type and mutant form of TGF‐β1's promoter. E, Knockdown of c‐Jun suppressed the activity of WT TGF‐β1's promoter but failed to affect MUT TGF‐β1's promoter. F, MUC12 promoted the recruitment of c‐Jun to TGF‐β1's promoter. G, MUC12 increased the activity of WT TGF‐β1's promoter but failed to affect MUT TGF‐β1's promoter. *P < 0.05; **P < 0.01

Next, we want to investigate whether MUC12 relies on c‐Jun's transactivating ability to regulate TGF‐β1. We first confirmed that c‐Jun depletion by siRNAs could reduce TGF‐β1 at both protein and mRNA level (Figure 6G,H). By analysing the promoter region of TGF‐β1 using c‐Jun as a bait, we identified there were four potential DNA responsive elements of c‐Jun (Figure 7A,B). To end this, we designed two pairs of primers (c‐JUNE1 and c‐JUNE2) as it is difficult to separately analyse them, to examine whether c‐Jun directly binds to TGF‐β1's promoter. ChIP assay result indicated that c‐Jun could bind to c‐JUNE2 but not c‐JUNE1 promoter region of TGF‐β1 (Figure 7C). Next, we constructed 2 kb wild‐type promoter region of TGF‐β1 (WT) as well as the c‐JUNE2 mutated form (MUT) into pGL3 to investigate whether MUC12 and c‐Jun could regulate its activity (Figure 7D). As expected, depletion of c‐Jun could suppress the WT activity but fail to affect it when c‐JUNE2 was mutated (Figure 7E). Importantly, MUC12 could promote the recruitment of c‐Jun to the promoter region of TGF‐β1 (Figure 7F). Similarly, MUC12 could enhance the promoter activity of WT TGF‐β1 but lost this ability when the WT was replaced by MUT (Figure 7G). All these results demonstrate that MUC12 relies on the transactivation of c‐Jun to activate TGF‐β1, leading to RCC progression.

## 4 DISCUSSION

RCC remains the most common kidney cancer.^3^ Although current therapeutic treatments benefit RCC patients a lot, the survival rate of metastatic RCC is still very low. Thus, there is an urgent need of developing novel therapies for better treatment of RCC. In this study, we found that MUC12 was overexpressed in RCC patients compared to normal kidney tissues and its levels were gradually increased as this type of cancer progressed to later stage according to TCGA data set. In vitro experimental results also confirmed that MUC12 served as tumour‐promoting factor to increase RCC cell growth and cell invasion. Mechanistically, MUC12 activated TGF‐β1 signalling dependent of the transcriptional regulation of TGF‐β1 by c‐Jun. Inhibition of TGF‐β1 signalling could partially reverse biological effects of MUC12 on RCC cells. Overall, our data define the role of MUC12 in RCC progression and build rational to develop MUC12 targeted therapy for RCC patients.

MUC12 is membrane glycoprotein and its biological contributions to cells may rely on signal transduction cassette. Here, our data exhibited that MUC12 bore the ability to increase c‐Jun protein levels, which in turn transcriptionally regulated TGF‐β1. Given the fact that c‐Jun is a transcription factor working in the nucleus, the process by which MUC12 regulates c‐Jun has a spatial distance. We hypothesize that MUC12 may serve as scaffold protein to activate certain kinase cassette, which phosphorylates and activates c‐Jun. Previous reports have demonstrated that the phosphorylation of c‐Jun in its N‐terminal by ERK or JNK could increase its stability and enhance its DNA binding ability.^22^, ^23^ Interestingly, both ERK and JNK have change to be activated by membrane proteins such as G‐protein receptor, which suggests that MUC12 also has chance to activate them. Nevertheless, the detailed mechanisms of how MUC12 activates c‐Jun deserve future intensive investigations.

As a membrane protein, MUC12 has a promising therapeutic potential. Recently, chimeric antigen receptor (CAR) T cell therapy has been merged as one alternative therapy for multiple cancers.^24^ CAR‐T cells with gene modification can express chimeric receptor and specifically bind to tumour antigen, leading to specific cytotoxicity of T cells towards cancer cells. However, CAR‐T cell therapy in advanced RCC is still on its infancy. The property of MUC12 in metastatic RCC makes it a promising target for CAR‐T cells. In fact, MUC family member has been viewed as ideal target for CAR‐T cells in various cancers: CAR‐T cell therapy specific for MUC1 has been clinically tested in HCC, breast and glioma ^25^; CAR‐T cells specific for MUC16 have also being investigated in ovarian cancer.^26^ Thus, it is also reasonable to construct specific CAR‐T cells to kill MUC12‐positive advanced RCC.

TGF‐β signalling is widely known to promote cancer development. In one hand, TGF‐β signal can promote the epithelial to mesenchymal transition (EMT) of cancerous cells, which bestows cancer cells metastatic potential and allows them to move to distant organs.^20^ In another hand, TGF‐β signalling also can educate tumour microenvironment and make it suitable for cancer survival.^27^ For instance, the transformation of fibroblasts into myofibroblasts (so‐called cancer‐associated fibroblasts or CAFs) is accelerated in the presence of TGF‐β.^27^ These CAFs play critical role in tumour survival and cancer metastasis. Due to the importance of TGF‐β in cancer progression, now several inhibitors are being tested in clinical trials including metelimumab, fresolimumab and AVID200.^28^ Our study also pointed out that TGF‐β1 was involved in MUC12‐mediated cell invasion, strengthening the role of TGF‐β1 signalling in the development of advanced RCC.

In summary, our data identify MUC12 as a tumour promoting factor in advanced RCC by activating TGF‐β1 signalling and provide compelling rational to develop MUC12‐targeted therapies for RCC patients.

## CONFLICT OF INTEREST

This study has no potential conflict of interest to declare.

## AUTHOR CONTRIBUTION

**Shenglin Gao:** Conceptualization (lead); Data curation (lead); Methodology (lead); Resources (equal); Software (equal); Supervision (equal); Validation (equal); Visualization (equal); Writing‐original draft (lead); Writing‐review & editing (lead). **Rui Yin:** Conceptualization (equal); Data curation (equal); Project administration (equal); Writing‐original draft (equal); Writing‐review & editing (equal). **Li‐Feng Zhang:** Data curation (equal); Funding acquisition (equal); Project administration (equal); Resources (equal); Software (equal). **Si‐Min Wang:** Formal analysis (equal); Investigation (equal); Methodology (equal); Resources (equal); Software (supporting); Validation (supporting). **Jiasheng Chen:** Formal analysis (equal); Software (supporting); Supervision (supporting). **Xing‐Yu Wu:** Methodology (supporting); Project administration (supporting); Software (supporting); Supervision (supporting). **Chuang Yue:** Methodology (supporting); Project administration (supporting); Resources (supporting); Software (supporting). **Li Zuo:** Conceptualization (equal); Data curation (lead); Formal analysis (equal); Funding acquisition (lead); Resources (equal); Writing‐original draft (lead); Writing‐review & editing (lead). **Min Tang:** Data curation (equal); Software (lead); Validation (lead); Writing‐original draft (equal); Writing‐review & editing (equal).

## Supporting information

Supplementary Material

## Data Availability

The data sets used and/or analysed during the current study are available from the corresponding author on reasonable request.
