# Supplementary material for: The oncogenic role of MUC12 in RCC progression depends on c‐Jun/TGF‐β signalling
Source: J Cell Mol Med. 2020 Jun 28;24(15):8789–802. doi: 10.1111/jcmm.15515 (PMC7412406; doi:10.1111/jcmm.15515)
Supplement: Supplementary file 1 — Supplementary Material [file JCMM-24-8789-s001.docx]

**The** **oncogenic role of MUC12 in RCC progression depends on c-Jun/TGF-β1 signaling**

**Author list:** Sheng-Lin Gao^1 #^, Rui Yin ^2 #^, Li-Feng Zhang^1 #^, Si-Min Wang^3^, Jia-Sheng Chen^1^, Xing-Yu Wu^1^, Chuang Yue^1^, Li Zuo^1 *^, Min Tang^4 *^

**Institution:**

**^1^**Department of Urology, The Affiliated Changzhou No. 2 People’s Hospital of Nanjing Medical University, Changzhou, Jiangsu, China

**^2^**Center for Reproductive Medicine, Shandong Provincial Hospital Affiliated to Shandong University, Jinan, Shangdong, China

^3^Changzhou Third People's Hospital, Changzhou, Jiangsu, China

^4^Department of Urology，The First Affiliated Hospital of Nanjing Medical University, Nanjing, Jiangsu, China

^#^These authors contributed equally to this work.

^*^Correspondence to:

**Name:** Li Zuo(first corresponding author)

**Address:** Department of Urology, The Affiliated Changzhou No. 2 People’s Hospital of Nanjing Medical University, Changzhou, Jiangsu, China

**Post code:** 213000

**Telephone:** (+86)15051957700

**Fax:** 0519-88104931

**Email:** [zuoli@njmu.edu.cn](mailto:zuoli@njmu.edu.cn)

**Name:** Min Tang

**Address:** Department of Urology，The First Affiliated Hospital of Nanjing Medical University, Nanjing, Jiangsu, China

**Post code:** 210000

**Telephone:** (+86)13621580823

**Fax:** 025-68303171

**Email:** mintang@njmu.edu.cn

**Supplemental figure**


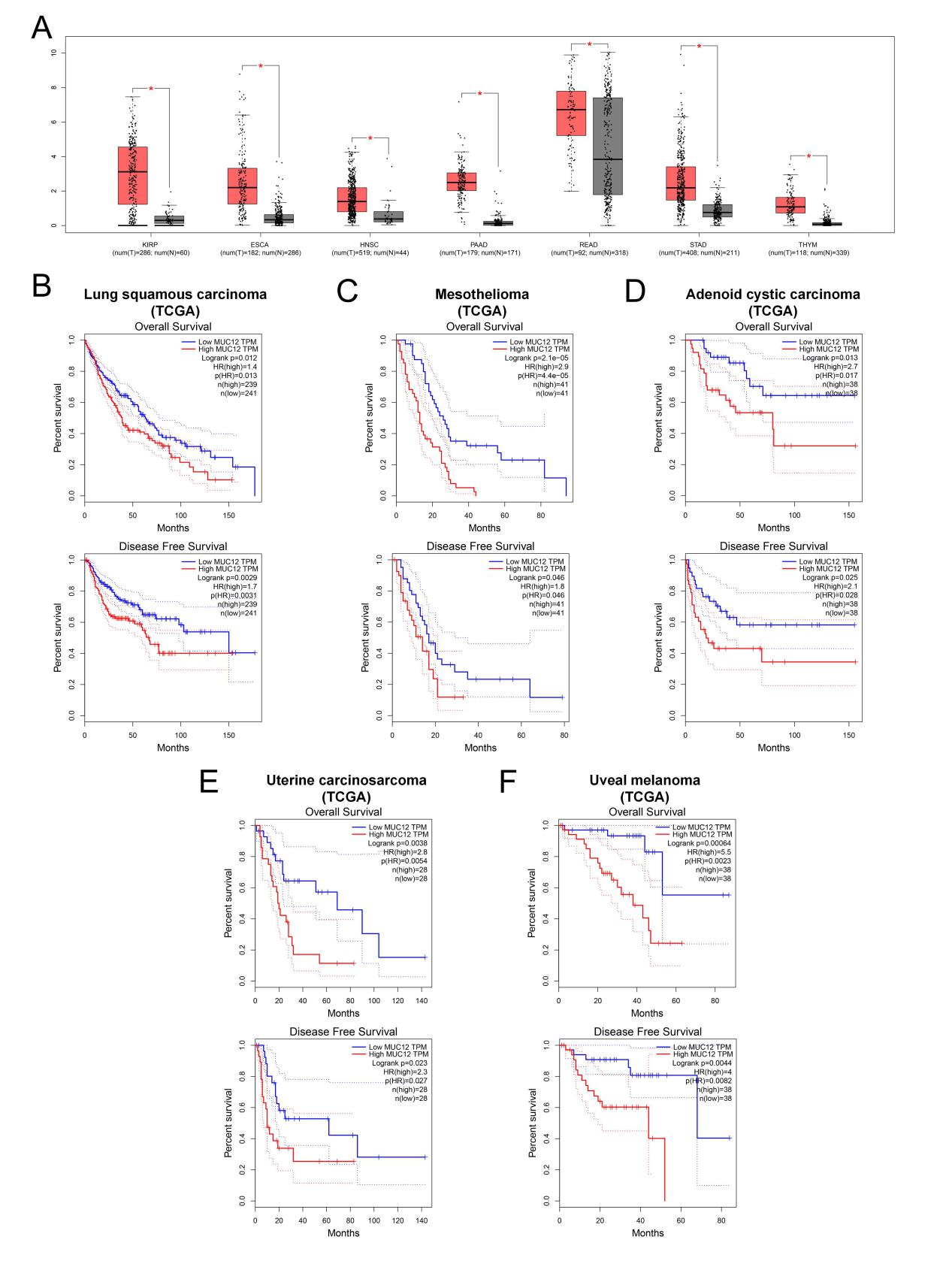


**Supplementary Fig1.** MUC12 was overexpressed in other various types of human cancer. **A**. MUC12 was significantly high expression in kidney renal papillary cell carcinoma, esophageal carcinoma, pancreatic adenocarcinoma, rectum adenocarcinoma, stomach adenocarcinoma, thymoma, head and neck squamous cell carcinoma. B-F. MUC12 overexpression correlated with poor prognosis in lung squamous carcinoma, mesothelioma, adenoid cystic carcinoma, uterine carcinosarcoma and uveal melanoma. * *p*<0.05.

**Supplemental Tables**

**Table S1. Comparison of baseline clinicopathological characteristics based on TCGA**

|  |  | TCGA Cohort (n= 533) | |  |
| --- | --- | --- | --- | --- |
|  | Cases No. | MUC12 | | P |
|  |  | Low | High |  |
| **Age (years)** |  |  |  |  |
| ≤60 | 264 | 113 | 151 | 0.114 |
| >60 | 268 | 133 | 135 |  |
| NA | 3 |  |  |  |
| **Gender** |  |  | |  |
| Male | 345 | 166 | 179 | 0.218 |
| Female | 188 | 80 | 108 |  |
| NA | 2 |  |  |  |
| **Pathology_T_stage** |  |  | |  |
| T1-2 | 342 | 141 | 201 | 0.002** |
| T3-4 | 191 | 105 | 86 |  |
| NA | 2 |  |  |  |
| **Pathology_N_stage** |  |  | |  |
| N(-) | 240 | 111 | 129 | 0.008** |
| N(+) | 16 | 13 | 3 |  |
| NA | 279 |  |  |  |
| **Pathology_M_stage** |  |  | |  |
| M (-) | 422 | 176 | 246 | <0.001*** |
| M (+) | 79 | 53 | 26 |  |
| NA | 34 |  |  |  |
| **Grade** |  |  | |  |
| G1-2 | 243 | 78 | 165 | <0.001*** |
| G3-4 | 282 | 163 | 119 |  |
| NA | 10 |  |  |  |
| **Cancer_Status** |  |  | |  |
| Recurrence (-) | 360 | 140 | 220 | <0.001*** |
| Recurrence (+) | 139 | 88 | 51 |  |
| NA | 36 |  |  |  |
| **Laterality** |  |  | |  |
| Left | 251 | 126 | 125 | 0.083 |
| Right | 281 | 120 | 161 |  |
| Bilateral | 1 | 0 | 1 |  |
| NA | 2 |  |  |  |

* Statistically significant (***p*<0.01, ****p*<0.001); *p* value from Cox regression analyses

**Table S2. Univariate and multivariate cox regression analyses of MUC12 expression and overall cancer survival in patients with RCC**

|  | Univariate analysis | Multivariate analysis |
| --- | --- | --- |
|  | HR (95% CI) *P* Value | HR (95% CI) *P* Value |
| MUC12 expression |  |  |
| Low | 1.0 (Reference) | 1.0 (Reference) |
| High | 2.399(1.753-3.284) *<0.0001**** | 1.542(1.110-2.143) *0.01** |
| Age |  |  |
| <60 | 1.0 (Reference) | 1.0 (Reference) |
| ≥60 | 1.740(0.880-2.365) 0.777 | NA |
| Gender |  |  |
| Male | 1.0 (Reference) | 1.0 (Reference) |
| Female | 1.101(0.808-1.500) 0.543 | NA |
| Tumor side |  |  |
| Left | 1.0 (Reference) | 1.0 (Reference) |
| Right | 1.129(0.858-1.732) 0.270 | NA |
| Cancer status |  |  |
| Recurrence(-) | 1.0 (Reference) | 1.0 (Reference) |
| Recurrence(+) | 5.135(3.707-7.113) *<0.0001**** | 3.058(2.051-4.562) *<0.0001**** |
| T stage |  |  |
| T1–T2 | 1.0 (Reference) | 1.0 (Reference) |
| T3–T4 | 2.752(2.007-3.774) *<0.0001**** | 0.778(0.420-1.441) 0.425 |
| N stage |  |  |
| N(-) | 1.0 (Reference) | 1.0 (Reference) |
| N(+) | 5.135(3.707-7.113) *<0.0001**** | 3.058(2.051-4.562) *<0.0001**** |
| Grade |  |  |
| G1-2 | 1.0 (Reference) | 1.0 (Reference) |
| G3-4 | 2.422(1.706-3.438) *<0.0001**** | 1.431(0.977-2.096) 0.065 |
| Metastasis |  |  |
| M(-) | 1.0 (Reference) | 1.0 (Reference) |
| M(+) | 4.470(3.243-6.162) *<0.0001**** | 1.346(0.879-2.061) 0.171 |

CI confidence interval, HR hazard ratio

* Statistically significant (**p*<0.05, ***p*<0.01, ****p*<0.001)

*p* value from Cox regression analyses

| **Table S3. Primers information used in current work.** | | |
| --- | --- | --- |
| **Assay** | **Name** | **Sequence (5′→3′)** |
| Real-Time PCR | MUC12-F | CCTGGAAACCTTAGCACCAG |
|  | MUC12-R | GACACGCATTGTTTTCCAT |
|  | TGFB1-F | GGATACCAACTATTGCTTCAGCTCC |
|  | TGFB1-R | AGGCTCCAAATATAGGGGCAGGGTC |
|  | c-Jun-F | GGGAGCATTTGGAGAGTCCC |
|  | c-Jun-R | TTTGCAAAAGTTCGCTCCCG |
|  | E-cadherin-F | GGTCTGTCATGGAAGGTGCTC |
|  | E-cadherin-R | CAGGATCTTGGCTGAGGATGG |
|  | N-cadherin-F | TCAACTGCAACCGTGTCTGT |
|  | N-cadherin-R | ATCGATCTGGGTCCTGAGCA |
|  | Vimentin-F | TGGACCAGCTAACCAACGAC |
|  | Vimentin-R | GCCAGAGACGCATTGTCAAC |
|  | Snail-1-F | GCTCGAAAGGCCTTCAACTG |
|  | Snail-1-R | GACATGGCCTTGTAGCAGCC |
|  | ZO-1-F | CCCTCAAGGAGCCATTC |
|  | ZO-1-R | GAGTTTGCTCCAACGAGA |
|  | GAPDH-F | TCCAGAAACTAATGGCAGATCCC |
|  | GAPDH-R | AATTCCCTACGCTTTGGGTTTT |
| siRNA target sequence | c-Jun-si1 | GCCAACTCATGCTAACGCA |
|  | c-Jun-si2 | CAGCTTCCTGCCTTTGTAA |
|  | c-Jun-si3 | GCGCATGAGGAACCGCATT |
